# The electrical modulus and other dielectric properties by the impedance spectroscopy of LaCrO_3_ and LaCr_0.90_Ir_0.10_O_3_ perovskites

**DOI:** 10.1039/c7ra13261a

**Published:** 2018-01-25

**Authors:** M. Coşkun, Ö. Polat, F. M. Coşkun, Z. Durmuş, M. Çağlar, A. Türüt

**Affiliations:** Faculty of Engineering and Natural Sciences, Department of Engineering Physics, Istanbul Medeniyet University 34700 Üskudar Istanbul Turkey mustafa.coskun@medeniyet.edu.tr; Faculty of Engineering, Department of Industrial Engineering, Istanbul Kultur University 34156 Bakirkoy Istanbul Turkey o.polat@iku.edu.tr; Bağlar Mah., Gunesli Konutlar No: 38, D-24, 34212, Bagcilar Istanbul Turkey; Faculty of Science, Department of Physics, Anadolu University Eskisehir 26470 Turkey

## Abstract

We have prepared LaCrO_3_ (LCO) and 10% Ir doped LCO samples by the solid state reaction method and studied the electrical modulus and the other dielectric properties of the samples by means of the impedance spectroscopy in the −100 °C to 100 °C range, with steps of 20 °C. It has been clearly observed that the dielectric properties change due to Ir doping. The absolute dielectric constant value of Ir doped LCO has decreased and this reduction was attributed to decreasing Cr^6+^ ions which may play a vital role in space charge polarization and charge hopping. A plateau region appeared in the temperature-dependent real electrical modulus *M*′ *versus f* curves of the pure LCO sample while almost no plateau region is visible in the Ir doped LCO sample. The temperature-dependent imaginary modulus *M*′′ *versus f* curves has two peaks at each temperature; one of the peaks is at low frequency and the other at the high frequency region, which shifts through higher frequency region with increasing temperature. This originates from free charge accumulation at the interface with the increase of the temperature. Furthermore, it has been seen that the Ir doped LCO sample has higher impedance and resistance values than the undoped LCO sample at the same frequency and temperature. This phenomenon was attributed to doped Ir ions behaving like a donor in LCO because LCO is a p-type compound. Moreover, the activation energy values of 0.224 eV and 0.208 eV for LCO and of 0.161 eV and 0.265 eV for the Ir doped LCO have been obtained from the slopes of the *ρ*_dc_*vs.* (*kT*)^−1^ curves, respectively. Also the activation energies were calculated from the slopes of the *f*_max_*vs.* (*kT*)^−1^ curves and the obtained results from low frequency region were in good agreement with *ρ*_dc_*vs.* (*kT*)^−1^ ones.

## Abstract 2

In this work, LaCrO_3_ and Ir doped LaCrO_3_ compounds were synthesized and their frequency dependent electrical properties were studied.

## Graphical abstract

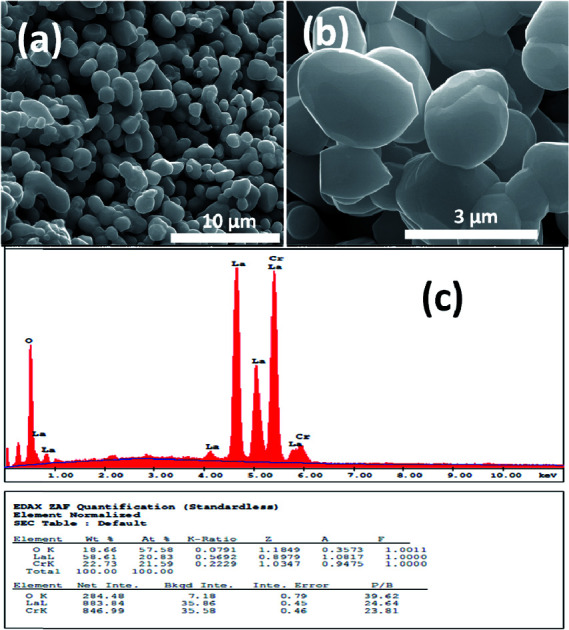

## 1. Introduction

The perovskite oxide compounds have the general stoichiometry of ABO_3_ formula in which A and B are cations and the A cation is larger than the B cation. The perovskite oxide materials have grabbed considerable attention among scientists due to their electrical, dielectric, magnetic, thermal, mechanical, sensing and optical properties.^1–7^ Additionally, the flexibility in the chemical composition of these materials offers excellent possibilities in terms of control of their structures *via* substitution of a number of transition metals into the A or B cation sites.^8–12^ Such properties make perovskite oxides very promising for several applications. The potential applications of these materials include ferroelectric random access memory, multilayer ceramic capacitors, magnetic field sensors, solid oxide fuel cells (SOFCs), membranes, catalytic converters^13–20^*etc.* Such wide range applications of these materials require us to understand their electrical and magnetic properties. Impedance spectroscopy is a well-developed tool to separate out the grain and grain boundary contributions to the total conductivity. Impedance technique acquires the electric response in a wide frequency range. The charge carrier in electric field could contribute to the electric response through motions such as charge displacement, dipole reorientation, space charge formation *etc.*^17–23^ The dielectric materials play an important role in the design of novel and high performances at electronic technologies. The dielectric, impedance, and modulus data often allow separately extracting additional useful information about the analysed dielectric materials. For instance, localized relaxations are calculated from the peaks at different frequencies in complex impedance or complex modulus *versus* frequency plots. The long-range fundamental conductivity results in exact overlapping of the modulus and impedance peaks.^24–26,29–34^ Especially, the investigation of electrical properties of these materials^27,28^ can provide us an insight for their application in sensors, optical memory, electro-optic devices and actuations in the micro-electromechanical systems *etc.*

LCO is one of those perovskite oxides has been studied extensively in the literature. LCO is a p-type compound with wide optical band-gap (3.4 eV), high electrical conductivity, high resistive to corrosive, high physical and chemical stability in ambient and has orthorhombic crystalline structure. The electrical, dielectric, magnetic and optical properties of LCO can be changed by doping both A (La) and B (Cr) cation sites with transition metals that offer excellent possibility to researchers to large application areas.^35–38^ The doped transition metal type has significant impact on the LCO properties because it changes almost all LCO parameter including physical and chemical. Many doping studies have been conducted on the LCO with various transition metals including Sr,^39^ Co,^40^ Ni,^41^ V,^41^ Cu,^41^ Mn,^42^ Ca,^43^ Pd^44^*etc.*

The aim of the present study is to investigate how transition element iridium, Ir, affects the electrical properties of LCO. It has been well documented that the ionic radius of the doped/foreign cations and their mixed-valences are found to be the key parameters governing the electrical and magnetic properties of the parent materials. Ionic radius of Ir varies depending upon oxidation states, for instance, the ionic radius of Ir^3+^ is 0.068 nm but the ionic radius of Ir^5+^ is 0.057 nm. Moreover, in the literature, it has been shown Ir may have +1 to +9 oxidation states.^45,46^ Therefore, we have been inspired from such features of Ir element to study the electrical properties of LCO compound when it is doped by Ir. As far as we are concerned electrical and dielectric properties of Ir doped LCO study has not been conducted in the literature, yet.

We have experimentally investigated the absolute dielectric constant |*ε*|, electrical modulus |*M*|, impedance |*Z*|, the resistivity *ρ*, real *M*′ and imaginary *M*′′ of electrical modulus by means of the impedance spectroscopy of LCO and Ir doped LCO perovskite samples. The samples have been prepared by the conventional solid state reaction technique. The dielectric parameters *versus* frequency measurements have been conducted from −100 °C to 100 °C range with steps of 20 °C. Moreover, the *ρ*_dc_*vs.* (*kT*)^−1^ and *f*_max_*vs.* (*kT*)^−1^ curves have also been plotted to obtain the activation energy values of both samples.

## 2. Experimental

The pure and Ir doped LaCrO_3_ powders were prepared *via* conventional solid state reaction technique. For pure LaCrO_3_ synthesis, La_2_O_3_ (ACROS, 99.9%) and Cr_2_O_3_ (ACROS, 99%) powders were used as starting materials. For the preparation of Ir doped LaCrO_3_ powders, IrO_2_ (ACROS, 99.99%), powders with mol% (10%) were mixed with La_2_O_3_ (ACROS, 99.9%) and Cr_2_O_3_ (ACROS, 99%) powders. For each powder the same synthesis steps were followed. Stoichiometric ratios of the mentioned powders were mixed in an agate mortar for 1 h with ethanol and calcined at 900 °C for 10 h in the air. The powders were then removed from the furnace and reground for homogeneity purpose. This step was pursued by the second calcination at 1200 °C for another 12 h in the air. Chemical stoichiometry of the powders was confirmed *via* Buker D8 Discover X-ray diffractometer (XRD) measurements. The XRD data of the synthesized powders have been presented in our previous study.^44^ Particle morphology of the synthesized powders was studied by a FEI scanning electron microscope (SEM) equipped with energy-dispersive X-ray spectrum (EDX). X-ray photoelectron spectroscopy (XPS) (SPECS) was used to characterize the surface of Ir doped LCO powder and bonding states of related elements using AlKα X-rays. In this analysis, the calibration was made according to the C 1s peak at 284.6 eV.

Pure and Ir doped LaCrO_3_ powders were pressed into 13 mm pellets at 10 tons pressure for 1 minute. After that, the pellets were gradually heated to 1150 °C for 4 h in air to sinter the targets. Novocontrol Broadband Dielectric/Impedance Spectrometer was employed for frequency depended electrical and dielectric property measurements. Temperature of the samples was gradually varied from −100 °C to 100 °C with 20 °C step.

## 3. Results and discussion

**Fig. 1:**
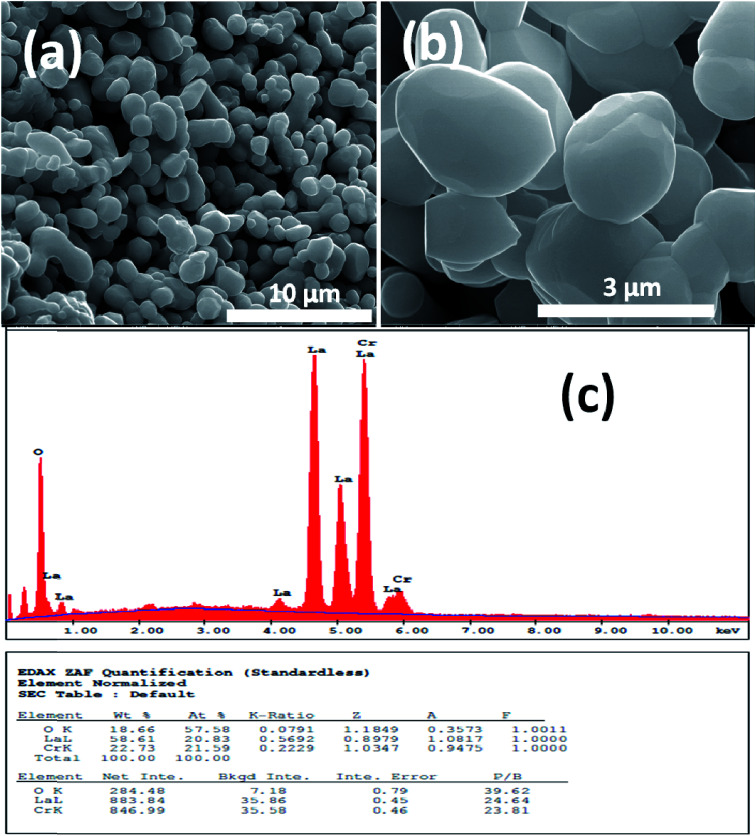
(a and b) SEM images with different magnification (c) EDX analysis results for the pure LaCrO_3_ powders, respectively.

**Fig. 2:**
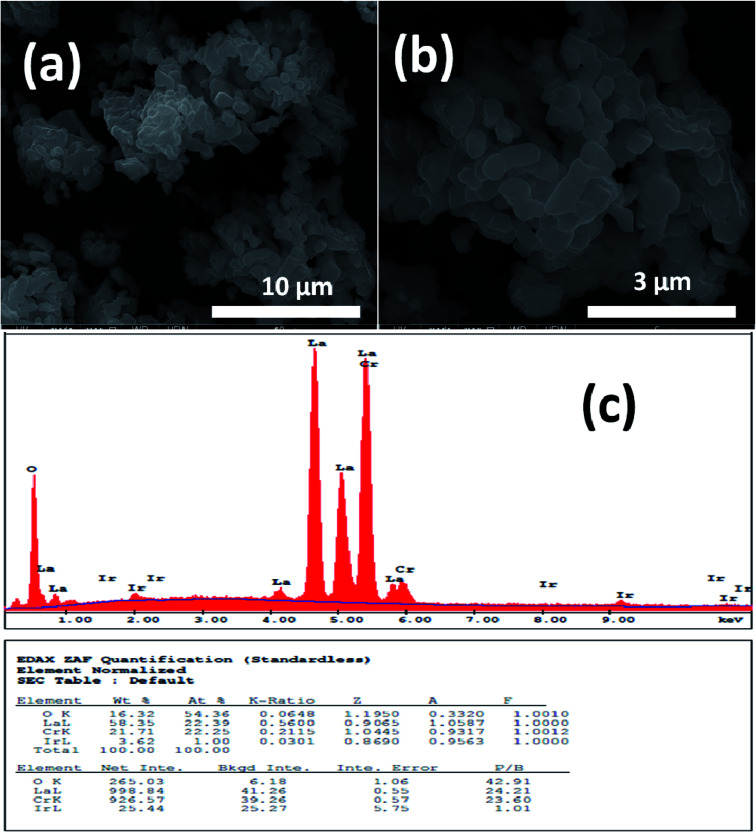
(a and b) SEM images with different magnification (c) EDX analysis results for the 10% Ir doped LaCrO_3_ powders, respectively.

Fig. 1 and 2 show (a) and (b) SEM images with different magnification (c) EDX analysis results for LCO and LaCr_0.90_Ir_0.10_O_3_ powders, respectively. As it can be seen from Fig. 1(a) and (b), LCO has spherical, oval and some irregular particle shapes. When it has been looked at Ir doped LCO powder in Fig. 2(a) and (b), the particles present non uniform morphologies. Moreover, images demonstrate the formation of cluster and agglomeration of particles. The energy-dispersive X-ray spectroscopy (EDX), a semi-quantitative elemental analysis technique, for the LCO and Ir doped LCO demonstrates in Fig. 1(c) and 2(c). EDX analysis has confirmed the presence of Ir in LCO sample.

**Fig. 3:**
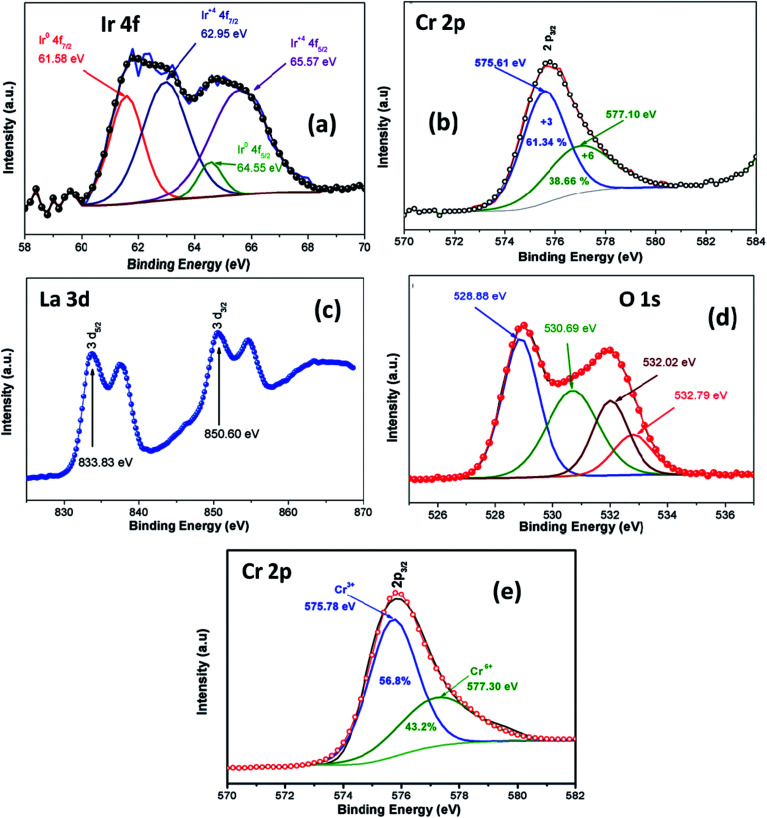
XPS analysis of (a) Ir 4f spectrum, (b) Cr 2p spectrum, (c) La 3d spectrum, (d) O 1s spectrum for Ir doped LCO and (e) Cr 2p spectrum for LCO.

XPS analysis was employed in order to investigate oxidation stage of Ir doped LCO powder and obtained results are presented in Fig. 3. As can be seen from Fig. 3(a) XPS analysis of Ir 4f was fitted into the two doublets (four peaks) due to the oxidized and metallic forms of Ir. The peaks at 61.58 eV and 64.55 eV are corresponding the metallic Ir^47^ whereas the peaks at 62.95 eV and 64.55 eV are due to the oxidized form of Ir (4+),^47^ respectively. Therefore, it can be said Ir has mixed oxidation states of 0 and 4+ in the doped LCO. The ratio of peak areas for 0 and 4+ valance states also calculated *via* Gaussian fitting, which are 28.1% for O oxidation and 71.9% for 4+ valance states. Oxidation of Cr atom in LCO and Ir doped LCO has been studied as well. The peak levels at 575.61 eV and 577.10 eV showed that Cr has mixed oxidation sates of 3+ and 6+ (2p_3/2_)^48^ in our studied Ir doped LCO sample and data are given in Fig. 3(b). The Gaussian fitting reveals the ratio of peak areas of Cr^3+^ as 61.34% and Cr^6+^ as 38.66% in the investigated sample. Also the peak levels at 575.78 eV and 577.30 eV showed that Cr has both 3+ and 6+ oxidation states for undoped LCO too and the result was given at Fig. 3(e) and the Gaussian fitting reveals that the ratio of peak areas of Cr^3+^ as 56.8% and Cr^6+^ as 43.2%. Moreover, valence state of La has been inspected. Two peaks at 833.83 eV (for 3d_5/2_) and 850.60 eV (for 3d_3/2_) reveal the valance state of La is 3+ in Ir doped LCO sample.^49^ Finally, we have performed XPS analysis on the oxygen oxidation state. O 1s spectra have been investigated for Ir doped LCO sample. The obtained data have been represented in Fig. 3(d). It can be seen that O 1s spectra have high energy tail (second peak around 532 eV). Such tail of O 1s spectra are often seen in oxides and perovskite-like compounds. Even though the origin of these peaks is subjected to a number of discussions,^50–55^ Begreuther *et al.* introduced new ideas to illuminate the O 1s high energy tails.^53^ They expressed that the tails may be related to (i) the various oxygen states which belong to different chemical bonds between oxygen–metal atoms, (ii) the defects within the crystal structure. In our studied Ir doped sample the presence of various oxidation states of Ir (metallic and 4+) and/or defects support Begreuther *et al.* ideas. Both EDX and XPS inspections have demonstrated that the transition element Ir has been doped into LaCrO_3_ structure and also any impurity has not been observed in the these analysis results. Further, any extra peak has not observed in XRD results that confirm our compounds do not have impurity.^44^

**Fig. 4:**
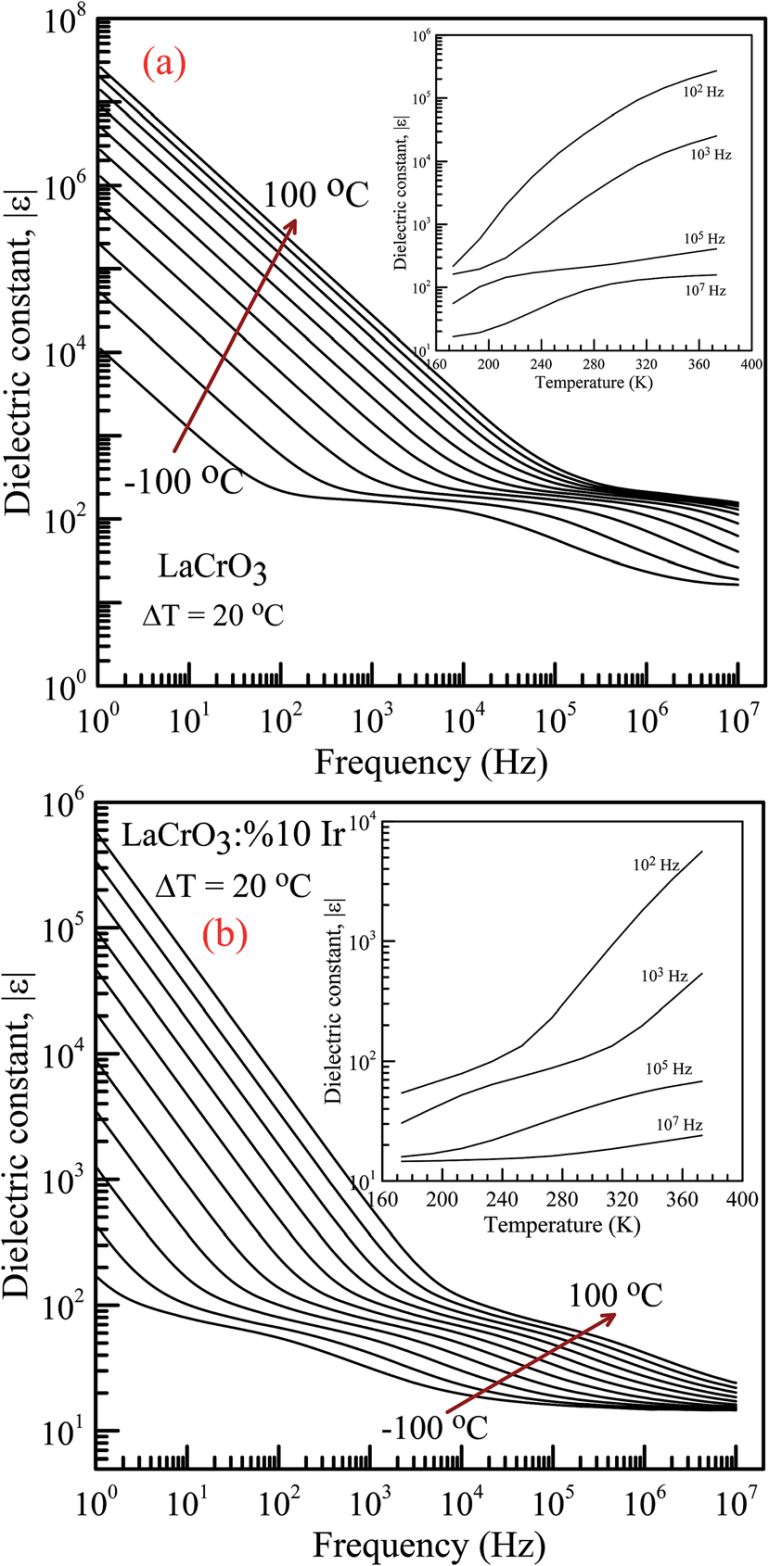
Frequency dependence of the dielectric constant |*ε*| in the temperature range from −100 °C to 100 °C with steps of 20 °C, (a) pure LaCrO_3_ and (b) LaCr_0.90_Ir_0.10_O_3_. Inset figures show |*ε*| *vs. T* at 10^2^, 10^3^, 10^5^ and 10^7^ Hz.

Fig. 4(a) and (b) represent the frequency (*f*) dependence of the dielectric constant |*ε*| in the temperature range from −100 °C to 100 °C with steps of 20 °C, (a) the pure LaCrO_3_ and (b) LaCr_0.90_Ir_0.10_O_3_, where |*ε*| is in terms of a magnitude (absolute value) of *ε* and the inset figures show temperature dependent dielectric constant |*ε*| with different frequency. As seen from Fig. 4(a) and (b), the dielectric constant increases with increasing temperature at a given frequency (see inset figures). At the frequency of 1.0 Hz, LCO sample has the *ε* values of approximately 10^4^ at −100 °C and 3 × 10^7^ at 100 °C, whereas 10% Ir doped LCO sample has the |*ε*| values of approximately 180 at −100 °C and 6 × 10^5^ at 100 °C. That is, LCO sample has higher *ε* value than the Ir doped LCO sample at a given frequency. We believe that such decrease in |*ε*| value might be related to reduction of the number of Cr^6+^ ions, which may play a vital role on space charge polarization and charge hoppings, in LCO parent material due to substitution of Ir^4+^ ions. Liu *et al.* have shown that Sr doping into La in LCO structure boosts the conductivity and dielectric constant due to the generation of more Cr^6+^ ions.^56^ The XPS analysis confirmed that even though Cr^3+^ concentration increases from 56.8% to 61.34% for LCO and Ir doped LCO, the Cr^6+^ concentration lowers from 43.2% to 38.66% (see Fig. 3). Furthermore, it has been observed the plateau in the dielectric constant *vs.* frequency curves of LCO sample at each temperature. For example, the plateau of the curve for LCO at −100 °C ranges from approximately |*ε*| = 180 at 10^2^ Hz to |*ε*| = 125 at 10^4^ Hz, and plateau of the curve at 100 °C begins from |*ε*| = 240 at 3 × 10^5^ Hz and beyond 10^7^ Hz. The frequency range of the plateau in the |*ε*|–*f* curves of LCO sample increases with increasing temperature again, the dielectric constant of LCO sample sharply decreases with increasing frequency to the starting point of the plateau at each temperature. Moreover, it can be seen from Fig. 4(a) and (b), the plateau for the doped LCO sample becomes more pronounced with increasing temperature while the plateau for the pure LCO sample has been observed at each temperature. The dielectric constant of LCO and Ir doped LCO samples sharply decreases with increasing frequency to a given frequency and then more slightly decreases to further frequencies at each temperature. The low and high frequency plateaus are attributed to extrinsic contributions from grain and grain boundary effects, respectively.

**Fig. 5:**
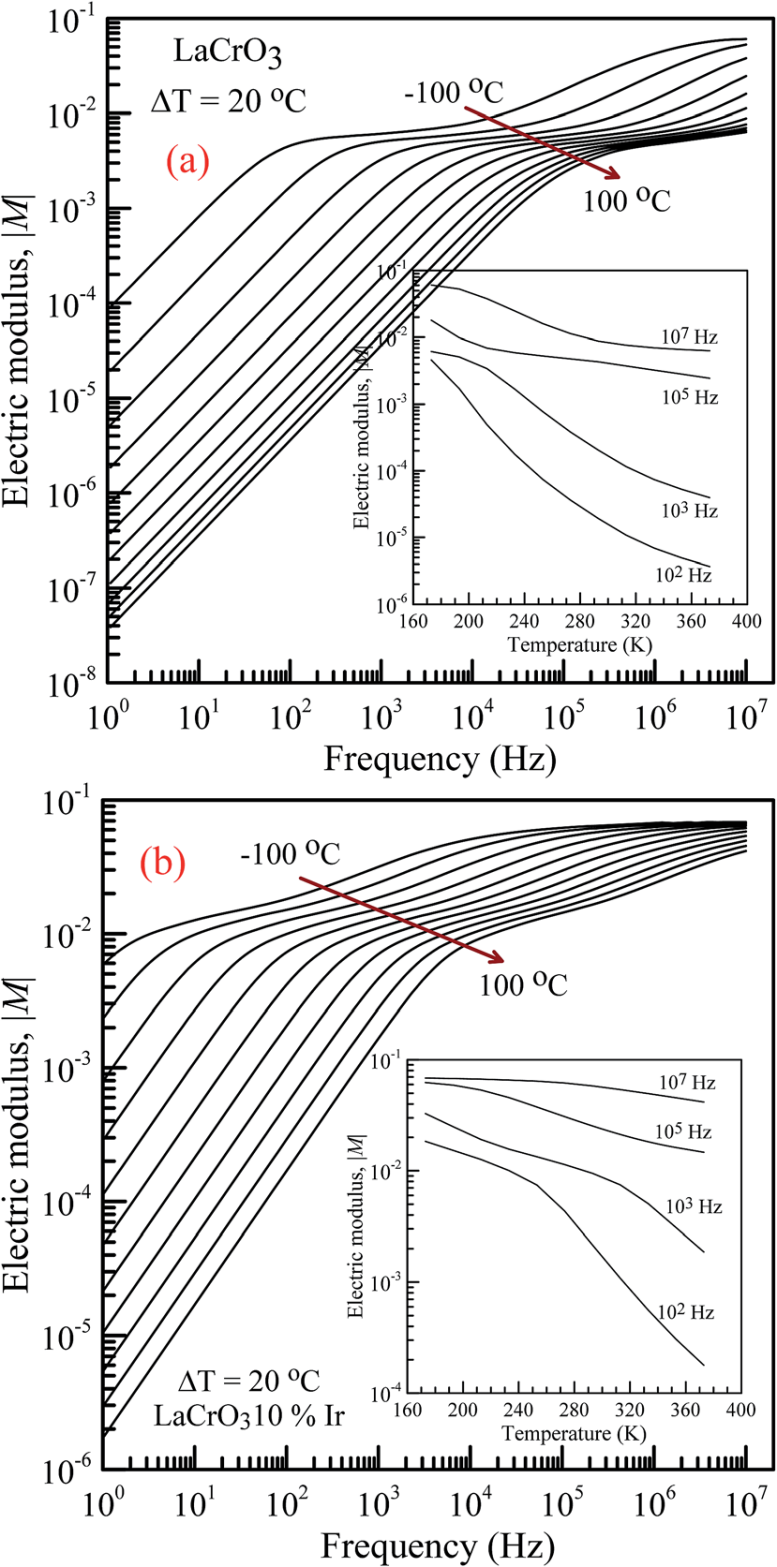
Frequency dependence of the electrical modulus of both samples in the temperature range from −100 °C to 100 °C with steps of 20 °C, (a) pure LaCrO_3_ and (b) LaCr_0.90_Ir_0.10_O_3_. Inset figures represent |*M*| *vs. T* at 10^2^, 10^3^, 10^5^ and 10^7^ Hz.

Fig. 5 shows the frequency dependence of the electrical modulus of (a) LCO and (b) Ir doped LCO samples, in the temperature range from −100 °C to 100 °C, where |*M*| is in terms of a magnitude (absolute value) of the electrical modulus *M* also the inset figures show electrical modulus *vs.* temperature with various frequencies, 10^2^, 10^3^, 10^5^ and 10^7^ Hz. The electric modulus physically corresponds to the relaxation of the electric field in the material, so that the electric modulus represents the real dielectric relaxation process. The complex electric modulus, M* = *M*′ + j*M*′′, is inversely proportional to permittivity of the material, M* = 1/ε*. The Complex modulus also provides an alternative approach (i) to analyze the electrical response of the materials and has been adopted by scientists to study relaxation phenomena in ceramics materials and ionic conductors,^17–24^ (ii) helps to confirm the ambiguity arising from the grain or grain boundary effect at elevated temperatures which may not be distinguished from complex impedance plots. The complex electric modulus has been discussed with both permittivity and impedance to study and analyze the contribution of the grain boundary on the relaxation mechanism of the materials also it offers us opportunities to investigate long-range conductions and localized dielectric relaxation phenomenon in microscopic level.^17–24^ The complex electric modulus is the inverse of complex permittivity and is given as follows:^17^1
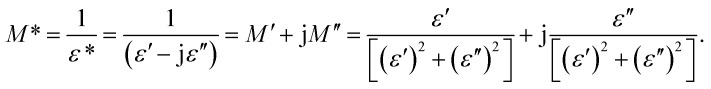


Moreover, it can also be expressed in terms of a derivative of complex impedance Z*.^17^

2 M* = *M*′ + j*M*′′ = j*wε*_0_Z* = j*wε*_0_*Z*′ − *wε*_0_*Z*′′

where *M*′ and *M*′′ are the real and imaginary parts of complex electric modulus M*, respectively, and *ω* = 2π*f* is a radial frequency expressed in radians/second parameter and is related to the applied ac frequency *f*.

**Fig. 6:**
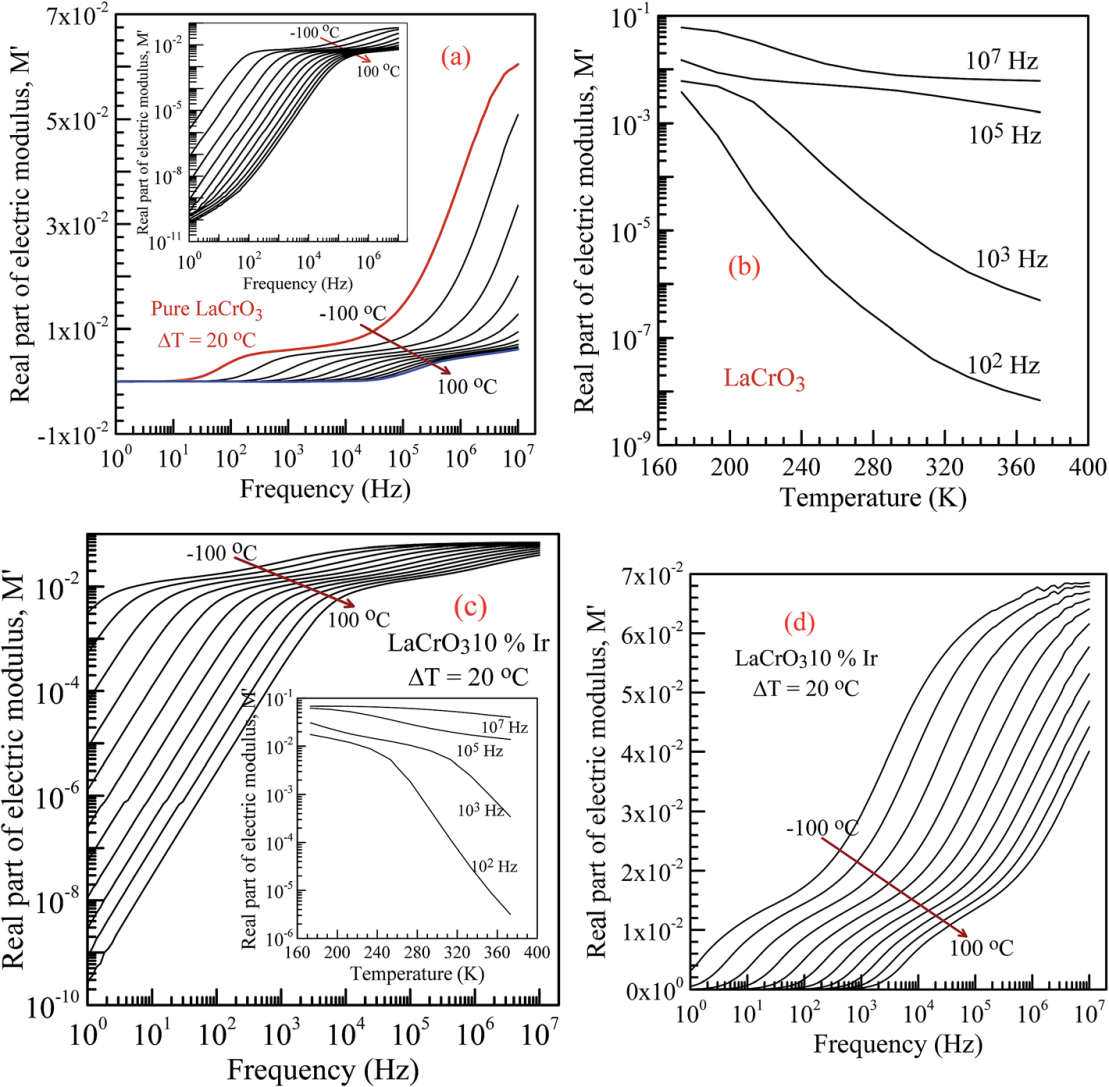
Frequency dependence plots of the real part of electrical modulus, *M*′ in the temperature range from −100 °C to 100 °C with steps of 20 °C, (a) the pure LaCrO_3_, inset shows its log–log plots of the real part of modulus with frequency (b) temperature *vs. M*′ plot for LCO at 10^2^, 10^3^, 10^5^ and 10^7^ Hz. (c) *M*′ *vs.* applied frequency for Ir doped LCO sample (inset figure shows temperature *vs. M*′ plot at 10^2^, 10^3^, 10^5^ and 10^7^ Hz) and (d) semi-log of the real part of modulus with frequency of the LaCr_0.90_Ir_0.10_O_3_.

Fig. 6 shows the frequency dependence curves of the real part of electrical modulus, *M*′, in the temperature range from −100 °C to 100 °C, (a) parent material LCO, inset shows the log–log plots of the real part of modulus with frequency; (b) shows temperature dependent real part of electrical modulus with different frequency of LCO, (c and d) display the log–log plots and semi-log of the real part of modulus of the Ir doped LCO as function of applied frequency and inset figure (c) shows temperature dependent real part of electrical modulus Ir doped LCO, respectively. When looking at the temperature-dependent *M*′ *versus f* curves of both samples, the *M*′ value increases with decreasing temperature at each frequency (see inset figures (b) and (c)), and it clearly seems that there is a difference in graphics due to the doped Ir. For example, in the log–log plots, the *M*′ *versus f* curve of LCO sample ranges from approximately 1.6 × 10^−6^ at 1.0 Hz to 6 × 10^−2^ at 10^7^ Hz for −100 °C, however, the curve of the Ir doped LCO varies from approximately 3 × 10^−3^ at 1.0 Hz to 7 × 10^−2^ at 10^7^ Hz for −100 °C. When we look at the applied temperature of 100 °C, the curve of LCO ranges from around 8 × 10^−11^ at 1.0 Hz to 6 × 10^−3^ at 10^7^ Hz, but the curve of the Ir doped LCO ranges from nearly 2.5 × 10^−10^ at 1.0 Hz to 4 × 10^−2^ at 10^7^ Hz. That is, the *M*′ *versus f* curve of the Ir doped LCO sample changes in a narrower range compared to that of the pure LCO at −100 °C. The change range expands with increasing temperature. As can be seen from the Fig. 6, the real part of electrical modulus, *M*′, of both samples increases with boosting frequency at a given temperature, and it reaches a constant value at high frequencies. Moreover, when we look at the temperature-dependent semi-log *M*′ *versus f* curves of both samples, Fig. 6(a) inset and (c), a plateau region seems in the *M*′ *versus f* curves of LCO sample whereas no plateau region almost is visible in those of the Ir doped LCO sample at each temperature.

**Fig. 7:**
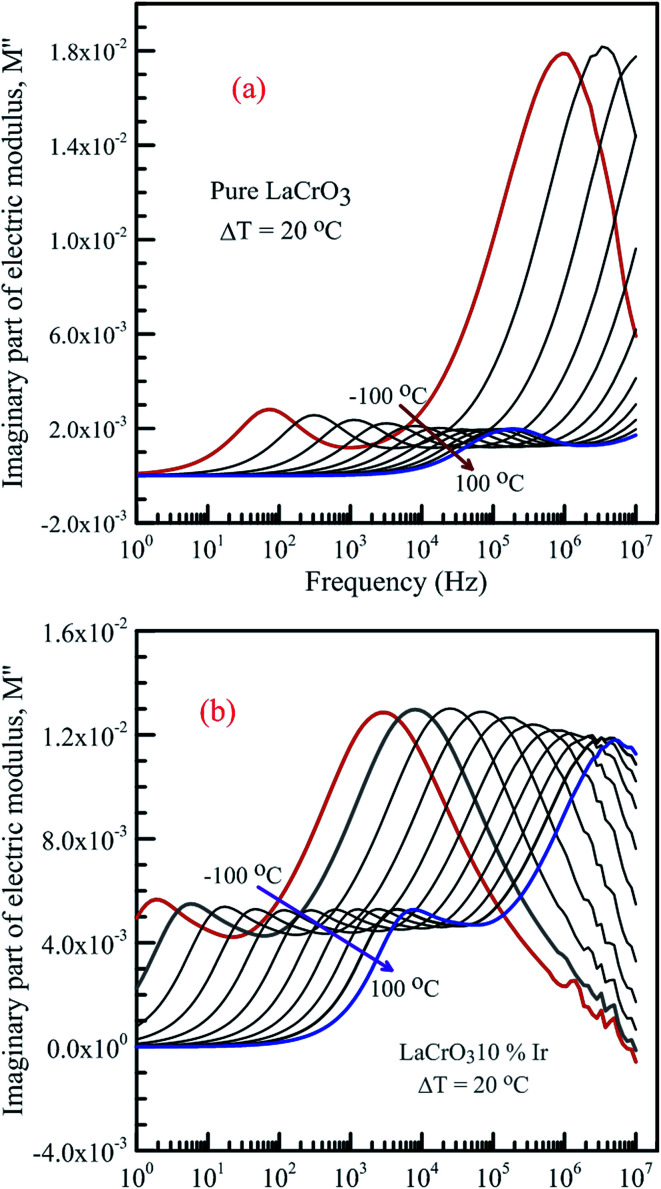
Frequency dependence plots of the imaginary part of electrical modulus, *M*′′ in the temperature range from −100 °C to 100 °C with steps of 20 °C, (a) pure LaCrO_3_ and (b) LaCr_0.90_Ir_0.10_O_3_.

Fig. 7 shows the frequency dependence curves of the imaginary part of electrical modulus, *M*′′ in the temperature range from −100 °C to 100 °C with steps of 20 °C, (a) LCO and (b) the Ir doped LCO, respectively. It is clearly seen that step like behavior was observed for both parent LCO and Ir doped LCO. It is clearly realized from Fig. 7(a) and (b) that *M*′′ *versus f* curves have two peaks at each temperatures, one of the peaks is at low frequency and other at high frequency region. It can be noticed that those peaks moves to higher frequency region *via* escalation of applied temperature. Such shift originates from free charge accumulation at the interface with the increase of the temperature. Thus, the increase in the charge carrier mobility decreases the relaxation time. The fact that the peaks shift to the higher frequency region with the increasing temperature indicates that dielectric relaxation is a thermally activated process in which the hopping mechanism of charge carriers dominates intrinsically. As can be seen from the figures, both peaks of the Ir doped LCO sample appears in the studied frequency range, 1.0 to 10 MHz. The second peak for parent LCO sample shifts towards frequencies over 10^7^ Hz at temperatures above −60 °C which is out of measurement frequency of our device. These peaks at low and high frequency regions demonstrate us that the mobility of ion migration from short time constant to long time constant passes *via* reducing frequency. The peak height in the *M*′′ *versus* frequency curves is proportional to capacitance, *C*^−1^, *i.e. M*′′_max_ = *C*_0_/2*C* and here *C*_0_ is the empty capacitance of sample.

**Fig. 8:**
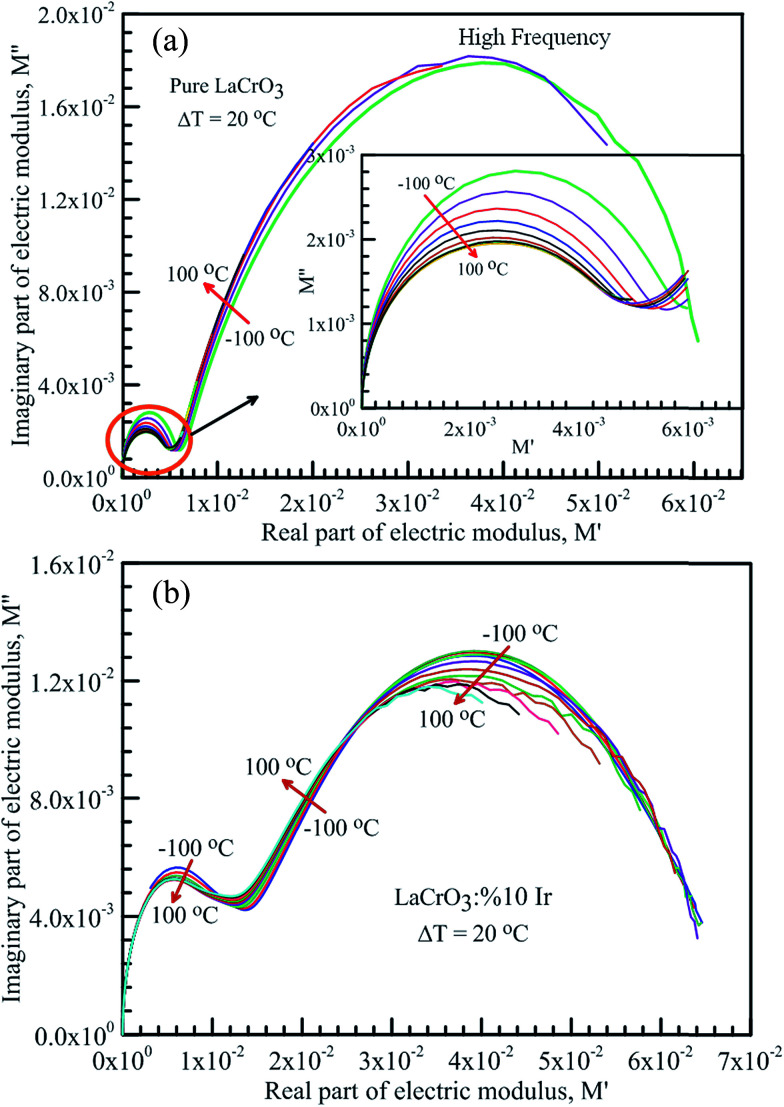
Frequency dependent-electrical modulus plane plots, the imaginary *M*′′ *versus* real *M*′ in the temperature range from −100 °C to 100 °C with steps of 20 °C, (a) pure LaCrO_3_, inset: low frequency data of the complex electric modulus plane plot of the pure LCO sample, and (b) LaCr_0.90_Ir_0.10_O_3_ sample.

Fig. 8 represents the frequency dependent-electrical modulus plane plots of both samples, the imaginary *M*′′ *versus* real *M*′ curves in the temperature range from −100 °C to 100 °C, (a) the parent LCO, inset: low frequency data of the complex electric modulus plane plot of LCO, and (b) the Ir doped LCO. The intercept on the real axis gives the capacitance from the grain or grain boundary. The semicircular arcs have been observed in the modulus plane plot of both samples in low and high frequencies, which points the presence of two relaxation processes. The small semicircular arcs at low frequency correspond to the contribution of weak grain boundary effects rather than the dominant grain effects. The large semicircular arcs at high frequency represent the bulk or grain response.^17–24,31^ A deviation from the semicircular shape of modulus arc of both samples has been observed with increasing temperature in high frequency region, and the semicircular arc for LCO sample has only formed at −100 and −80 °C, though the semicircular arc for the Ir doped LCO sample has only formed at the other frequencies except 60, 80 and 100 °C.

**Fig. 9:**
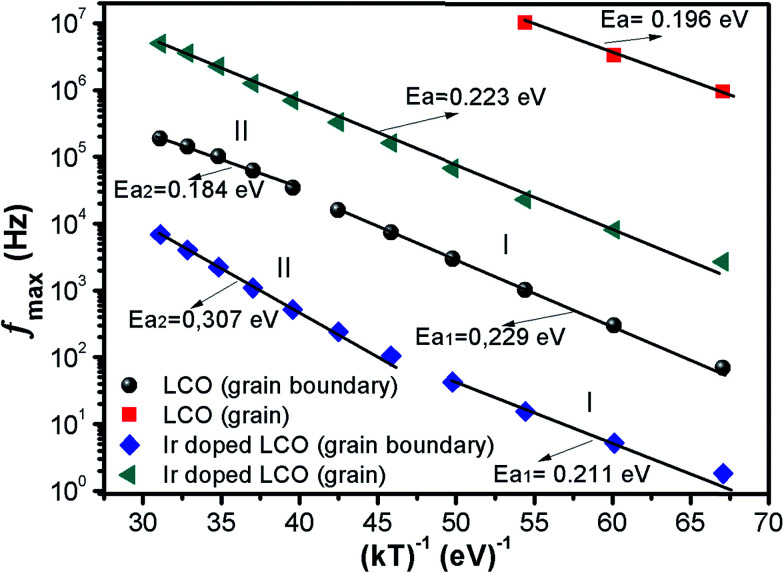
*f*_max_ *versus* (*kT*)^−1^ plots of both LCO and Ir doped LCO samples. The solid lines represents the fits to the experimental data.

The activation energy *E*_a_ of the LCO and Ir doped LCO samples have been calculated by using *M*′′ *vs.* frequency plots. The *M*′′ *vs.* frequency plots for LCO and Ir doped LCO have two peaks, as can be seen from the Fig. 7. In order to calculate activation energies an Arrhenius formula used that is given in eqn (3).3
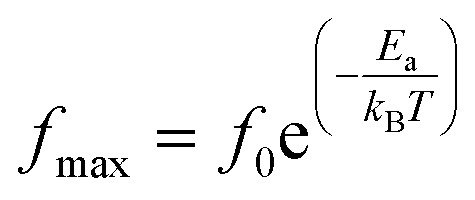
here frequency *f*_max_, at which the maximum *M*′′ takes place, *f*_0_ is a constant related to frequency, *E*_a_ is activation energy, *k*_B_ and *T* are Boltzmann constant and temperature in kelvin scale, respectively. It can be noticed from the Fig. 7 each compounds have two different (*f*_max_) that one corresponds for low frequency (grain boundary) and other originates higher frequency (grain) regions. The peak at low frequency region suggests that ions move over long distance while the peak at high frequency region proposes that ions move short distance due to confinement in their potential well.^57^ It can be seen from the Fig. 7 that the undoped LCO has just 3 relaxation peaks at higher frequency region (at −100, −80 and −60 °C) hence we have just 3 points in the graph. The activation energies of the LCO and Ir doped LCO compounds can be inferred from the slope of log(*f*_max_) *vs.* (*kT*)^−1^ plots, as exhibited in Fig. 9. The obtained *E*_a_ and *f*_0_ values are presented in Table 1. As can be seen from the Fig. 9 different activation energy values obtained from the slope of log(*f*_max_) *vs.* (*kT*)^−1^ plots. There are two different slope regions (I and II) at low frequency region (grain boundary) whereas only there is a single slope at high frequency region (grain) for both compounds. In our previous study, various activation energies have been attributed to different charge transportation mechanisms.^58^ The calculated activation energy values of 0.229 eV and 0.184 eV at regions I and II at low frequency (or grain boundary) regions and 0.196 eV at high frequency (or grain) region for LCO, 0.211 eV and 0.307 eV at regions I and II at low frequency (or grain boundary) regions and 0.223 eV at high frequency (or grain) region for Ir doped LCO obtained from the slope of log(*f*_max_) *vs.* (*kT*)^−1^ plots. The calculated activation energies for undoped and doped LCO are good agreement with other studies.^59,60^ The activation energies of Ir doped LCO higher than that of undoped LCO for all regions except region I (low frequency region). The increment of activation energy with Ir substitution to LCO might be explained by two factors: first may be attributed decreasing concentration of oxygen vacancies in the structure.^61^ LCO is the p-type perovskite when Ir^4+^ substitutes Cr^3+^, a positive charge forms at Ir site so Ir^4+^ behaves like a donor, and in order to maintain charge neutrality an electron will be created.^61^ This electron neutralizes the influence or contribution of hole. Consequently the concentration of oxygen vacancies diminishes and conductivity decreases or activation energy increases with doping Ir. Second might be ascribed to lattice distortion or defects owing to the Ir substitution into Cr. Our XPS data have exhibited the existence of Cr^3+^ (61.34%) (with ionic radius of 0.0615 nm) and Cr^6+^ (38.66%) (with ionic radius of 0.044 nm) mixed state in Ir doped LCO sample. Additionally, the same study has demonstrated Ir has also mixed oxidation states, Ir^4+^ (71.9%) (with ionic radius of 0.0625 nm) and Ir^0^ (28.1%) (with ionic radius of 0.18 nm). As it can be seen there is a significant distortion and strain can be expected in LCO lattice after Ir doped.

**Table 1:** The activation energy of LaCrO_3_ and Ir doped LaCrO_3_ from *f*_max_ & 1/*kT* plot

Material	*f*_max_ (Hz) (at low *f*)				*f*_max_ (Hz) (at high *f*)	
	*f*_0_ (Hz)		*E*_a_ (eV)		*f*_0_ (Hz)	*E*_a_ (eV)
	I	II	*E*_a1_	*E*_a2_		
LCO	2.28 × 10^8^	5.96 × 10^7^	0.229	0.184	4.44 × 10^11^	0.196
Ir doped LCO	1.52 × 10^6^	9.58 × 10^7^	0.211	0.307	5.22 × 10^9^	0.223

**Fig. 10:**
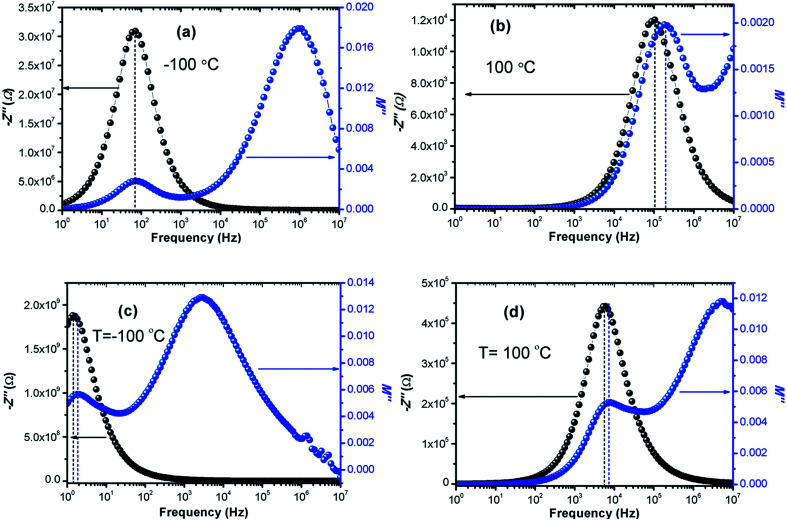
*Z*′′ and *M*′′ *vs.* frequency plots (a) −100 °C, (b) 100 °C for LCO and (c) −100 °C, (d) 100 °C for Ir doped LCO compounds.

The calculated activation energy values show that the hopping charged particles over the potential barrier are the origin of conduction mechanism for our compounds.^62^Fig. 10 shows the *Z*′′ and *M*′′ *vs.* frequency plots at −100 °C and 100 °C for LCO and Ir doped LCO compounds. It is noticed from these plots, the *Z*′′ has single peak whereas the *M*′′ has two peaks, one of them is at low frequency region other is at high frequency region. It can be seen from Fig. 10(a) the peak of *Z*′′ and *M*′′ (low frequency peak) *vs.* frequency occurs at same frequency at −100 °C for LCO that suggests a delocalized relaxation mechanism and having long-range mobility.^63^ On the other hand, this behavior has not been observed for LCO at 100 °C (Fig. 10(b)) also at −100 °C and 100 °C for Ir doped LCO (a slight mismatch can be clearly seen from Fig. 10(b–d)) which suggests localized relaxation mechanism of long-range interaction.^63^

**Fig. 11:**
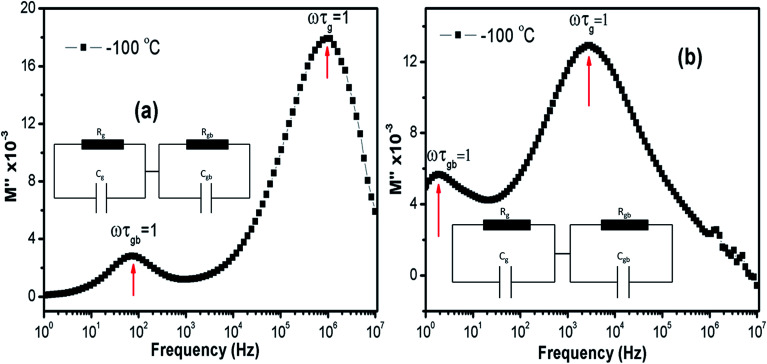
The *M*′′ *vs.* frequency behaviour for LCO and Ir doped LCO just at −100 °C and the relaxation points (*ω*_max_*τ* = 1). Inset figures show equivalent circuit for each compounds.

Relaxation time (*τ*) can be inferred using the formula *ω*_max_*τ* = (2π*f*_max_)*τ* = 1 in each peaks in the *M*′′ *vs.* frequency plots. Fig. 11(a) and (b) show the *M*′′ *vs.* frequency behavior for LCO and Ir doped LCO just at −100 °C and the relaxation points (*ω*_max_*τ* = 1) can be clearly seen for each compounds. I seen from Fig. 11 (inset figures) equivalent circuit composed from a two parallels RC elements that are connected in series. The relaxation time can be defined in terms of resistance (*R*) and capacitance (*C*) which are originating from grain and grain boundary as shown below:

4 *τ* = *RC*

5 *τ*_g_ = *R*_g_*C*_g_, *τ*_gb_ = *R*_gb_*C*_gb_

where *τ*_g_, *τ*_gb_, *C*_g_, *C*_gb_, *R*_g_ and *R*_gb_ relaxation time of grain, relaxation time of grain boundary, capacitance of grain, capacitance of grain boundary, resistance of grain and resistance of grain boundary respectively. The real and imaginary part of electrical modulus can be rewritten, in terms of parameters mentioned above, as shown below:6
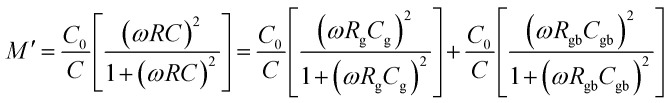
7
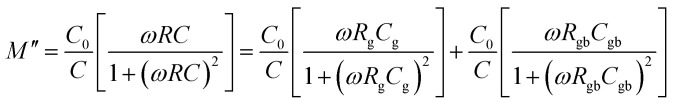


**Fig. 12:**
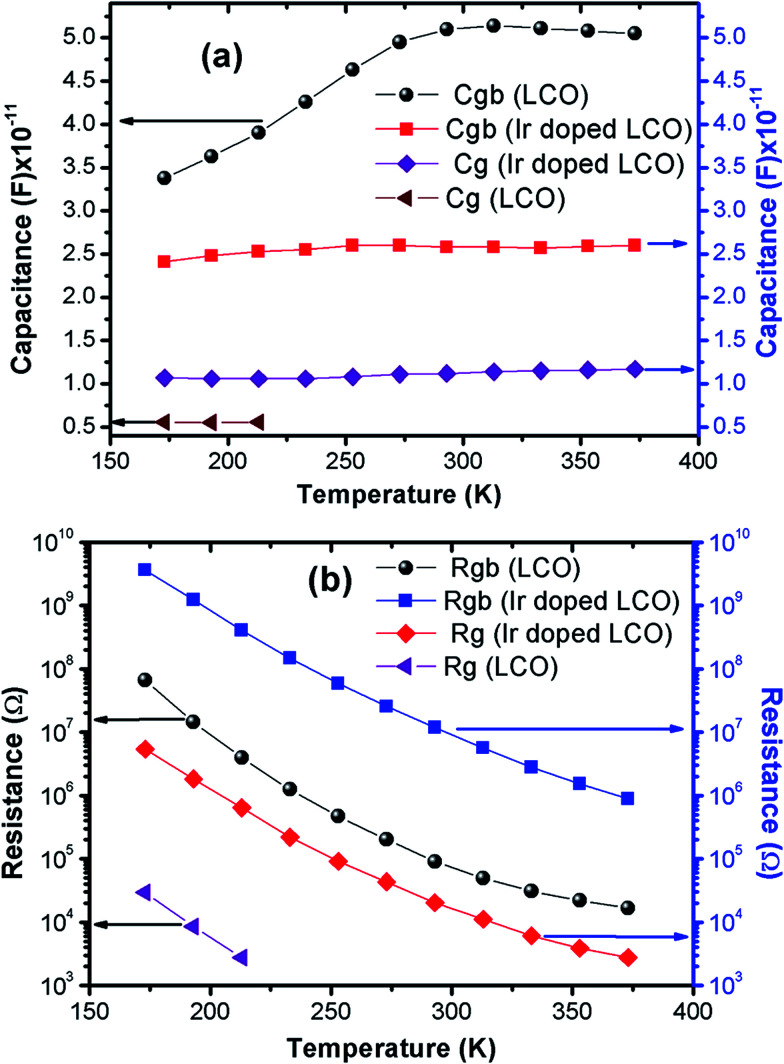
Temperature dependent (a) capacity and (b) resistance plots of grain and grain boundary for LCO and Ir doped LCO.

The capacitance values are determined using the *M*′′ *vs.* frequency plots due to the relation between *M*′′ = *C*_0_/2*C* at the relaxation points (*ω*_max_*τ* = 1) mentioned before. The calculated capacitance values for grain (*C*_g_) and grain boundary (*C*_gb_) are listed in Table 2. Now, we can calculate the resistance values for grain (*R*_g_) and grain boundary (*R*_gb_) using the eqn (5). The calculated *R* values were represented with together the capacitance values of *C*_g_ and *C*_gb_ in Table 2. Temperature dependent *C* and *R* plots are given in Fig. 12(a) and (b). It can be clearly seen that *C*_gb_ increases with increasing temperature and reaches a maxima then slightly decreasing with increasing temperature, for LCO. The *C*_g_ for LCO has just 3 points due to lack of relaxation points of the *M*′′ *vs.* frequency plots and as can be seen from the Fig. 12 (a) the *C*_g_ is the temperature independent. From the same figure, *C*_gb_ shows same behavior with the LCO one for Ir doped LCO and *C*_g_ is temperature independent at low temperature regions while slightly increasing with raising temperature at higher temperature regions. The temperature dependent both *R*_g_ and *R*_gb_ for both compounds show similar behavior decreasing with increasing temperature, as it is observed from Fig. 12(b). Furthermore, it is seen from the same figure, the *R*_gb_ is much greater than the *R*_g_ for both compounds that grain boundaries behave like insulator while grains behave like semiconductor.^64^

**Table 2:** The calculated relaxation time, capacity and resistance values of grain and grain boundary depend on temperature for LCO and Ir doped LCO

*T* (K)	LCO						Ir doped LCO					
	*τ*_gb_ (s)	*τ*_g_ (s)	*C*_gb_ (F) ×10^−11^	*C*_g_ (F) ×10^−12^	*R*_gb_ (Ω)	*R*_g_ (Ω)	*τ*_gb_ (s)	*τ*_g_ (s)	*C*_gb_ (F) ×10^−11^	*C*_g_ (F) ×10^−11^	*R*_gb_ (Ω)	*R*_g_ (Ω)
173	2.26 × 10^−3^	1.65 × 10^−7^	3.38	5.58	6.68 × 10^7^	2.96 × 10^4^	8.75 × 10^−2^	5.80 × 10^−5^	2.41	1.07	3.63 × 10^9^	5.42 × 10^6^
193	5.26 × 10^−4^	4.74 × 10^−8^	3.63	5.55	1.45 × 10^7^	8.55 × 10^3^	3.06 × 10^−2^	1.93 × 10^−5^	2.48	1.06	1.24 × 10^9^	1.83 × 10^6^
213	1.55 × 10^−4^	1.53 × 10^−8^	3.90	5.58	3.97 × 10^6^	2.74 × 10^3^	1.03 × 10^−2^	6.81 × 10^−6^	2.53	1.06	4.07 × 10^8^	6.43 × 10^5^
233	5.31 × 10^−5^		4.26		1.25 × 10^6^		3.75 × 10^−3^	2.33 × 10^−6^	2.55	1.06	1.47 × 10^8^	2.19 × 10^5^
253	2.18 × 10^−5^		4.63		4.70 × 10^5^		1.53 × 10^−3^	9.86 × 10^−7^	2.60	1.08	5.88 × 10^7^	9.10 × 10^4^
273	1.00 × 10^−5^		4.95		2.02 × 10^5^		6.66 × 10^−4^	4.82 × 10^−7^	2.60	1.11	2.56 × 10^7^	4.33 × 10^4^
293	4.62 × 10^−6^		5.10		9.05 × 10^4^		3.07 × 10^−4^	2.29 × 10^−7^	2.58	1.12	1.19 × 10^7^	2.04 × 10^4^
313	2.55 × 10^−6^		5.14		4.96 × 10^4^		1.46 × 10^−4^	1.26 × 10^−7^	2.58	1.14	5.66 × 10^6^	1.11 × 10^4^
333	1.58 × 10^−6^		5.11		3.09 × 10^4^		7.15 × 10^−5^	6.98 × 10^−8^	2.57	1.15	2.78 × 10^6^	6.09 × 10^3^
353	1.11 × 10^−6^		5.08		2.19 × 10^4^		3.95 × 10^−5^	4.47 × 10^−8^	2.59	1.16	1.53 × 10^6^	3.87 × 10^3^
373	8.48 × 10^−6^		5.05		1.68 × 10^4^		2.31 × 10^−5^	3.22 × 10^−8^	2.60	1.17	8.88 × 10^5^	2.76 × 10^3^

**Fig. 13:**
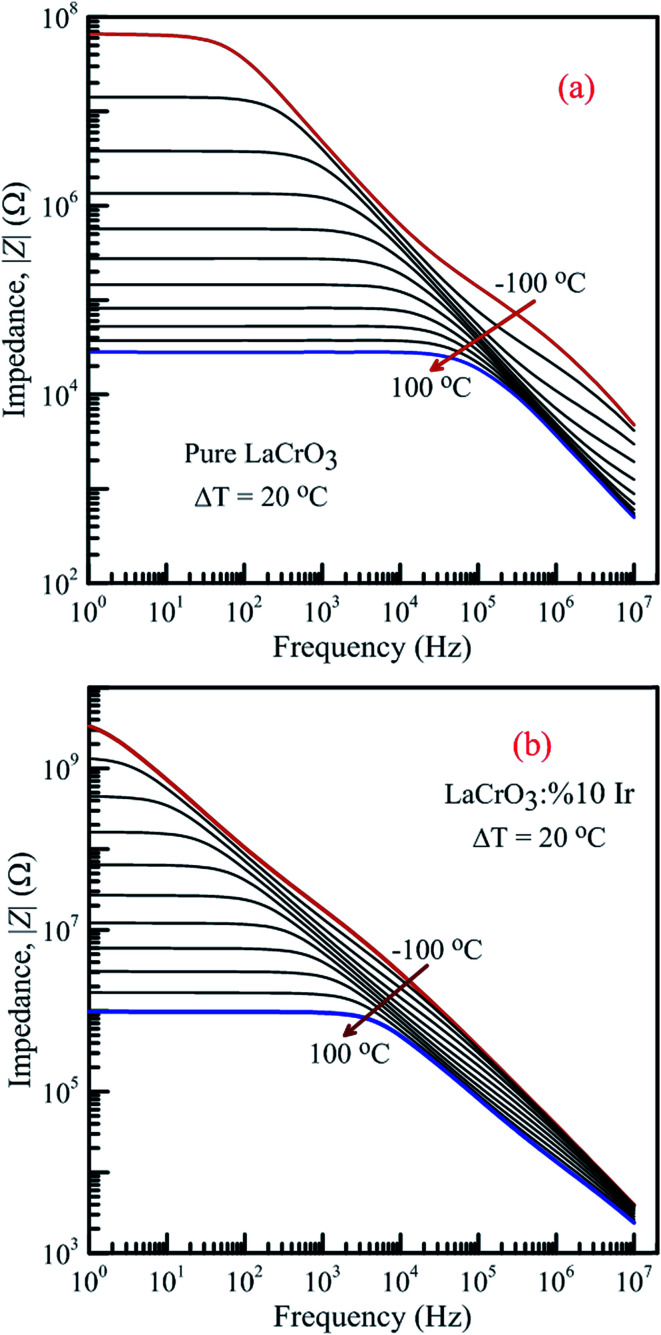
Frequency dependence of the impedance of both samples in the temperature range from −100 °C to 100 °C with steps of 20 °C, (a) pure LaCrO_3_ and (b) LaCr_0.90_Ir_0.10_O_3_.

Fig. 13 shows the frequency-dependent plots of the impedance of both samples in the temperature range from −100 °C to 100 °C, (a) LCO sample and (b) the Ir doped LCO sample, where |*Z*| is in terms of a magnitude (absolute value) of the impedance *Z*. Generally, the total impedance acts as pure capacitance at higher frequencies and pure resistance at lower frequencies. Fig. 13(a) and (b) exhibit that the impedance value of both samples increases with decreasing temperature at a given frequency. LCO sample has the values of about 6.5 × 10^7^ Ω at −100 °C and 3 × 10^4^ Ω at 100 °C, and the Ir doped LCO sample has the values of about 3.5 × 10^9^ Ω at −100 °C and 9.5 × 10^5^ Ω at 100 °C at low frequency region. That is, the Ir doped LCO sample has higher impedance value than LCO sample at each temperature. Such higher impedance value of Ir doped LCO is most likely attributed to strain and distortion of in LCO crystal structure due to substitution of Ir with larger ionic radius into smaller Cr^3+^ ion. The presence of distortion probably causes the reduction of the pathway of the mobility charge carriers. As a result, the impedance increases and conduction decreases in the doped LCO sample. It is known that LCO is a p-type compound when Ir^4+^ substitutes Cr^3+^, a positive charge forms at Ir site and in order to maintain charge neutrality an electron will be created so Ir^4+^ behaves like a donor in the LCO. Consequently, every created electron causes decreases conductivity or increases impedance of Ir doped LCO.

It is obvious from Fig. 13(a) and (b) the total impedance curves behave independent of frequency in low-frequency region and the frequency-dependent at high frequencies. The frequency range of the frequency-independent part of both samples decreases with increasing temperature. The frequency range of the frequency-independent part for LCO sample is wider than that of the Ir doped LCO sample at a given temperature.

**Fig. 14:**
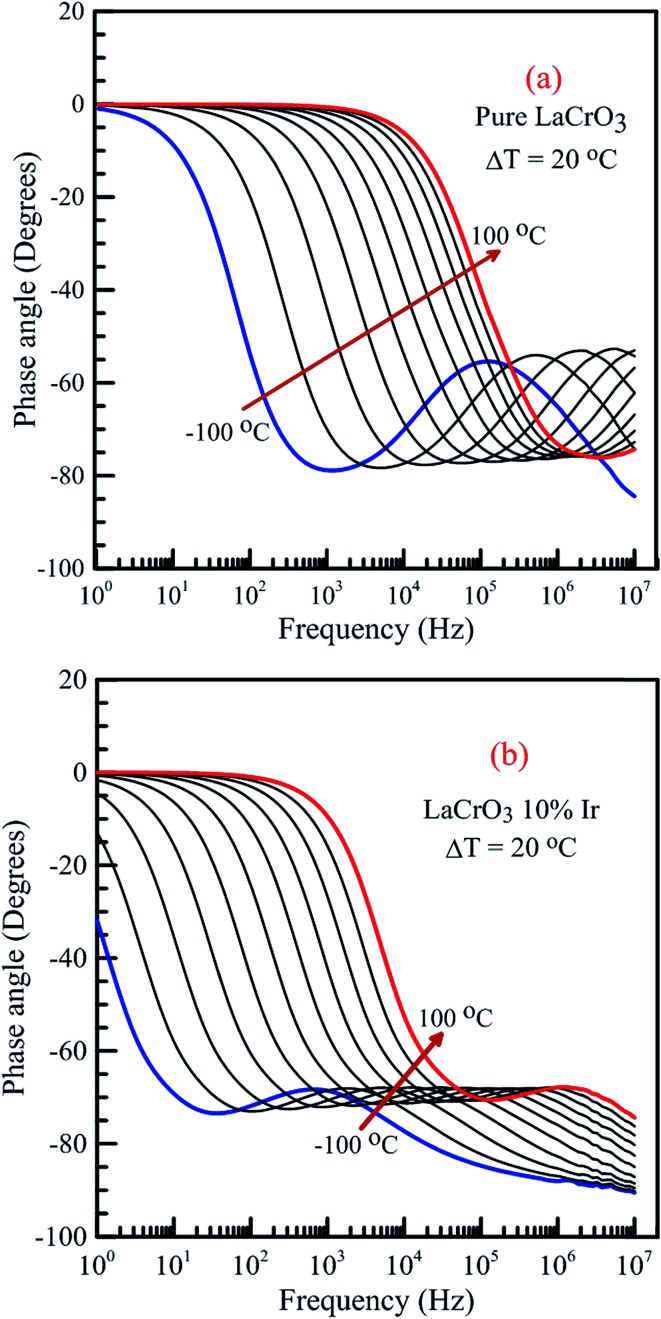
Frequency dependence of the phase angle of both samples in the temperature range from −100 °C to 100 °C with steps of 20 °C, (a) pure LaCrO_3_ and (b) LaCr_0.90_Ir_0.10_O_3_ samples.

Fig. 14 shows the frequency dependence curves of the phase angle in the temperature range from −100 °C to 100 °C, (a) LCO and (b) the Ir doped LCO samples. It can be realized from Fig. 14(a) and (b), both samples also have one peak each temperature. The peaks of the Ir doped LCO sample appear in the studied frequency range from −100 °C to 100 °C. Nevertheless, the peak of the pure LCO sample shifts towards frequencies over 10^7^ Hz at temperatures above −40 °C, that is, out of our measurement frequency range. For both samples, the peak shifts towards high frequencies with increasing temperature. Another difference appears in the temperature-dependent phase *versus* frequency curves of both samples. As can be from the these figures, as phase angle, the zero degree starting point for the curves of the parent sample corresponds to 1.0 Hz at all temperatures; but, for the Ir doped sample it only corresponds to 1.0 Hz for the curves above −40 °C, and shifts to lower frequencies than 1.0 Hz with decreasing temperature at temperatures below −40 °C. Generally, the phase angle tends to be −90° at high frequency that the impedance is completely capacitive, and to 0° at low frequency that it becomes completely resistive, that is, the impedance approaches the *R* value at low frequencies.

**Fig. 15:**
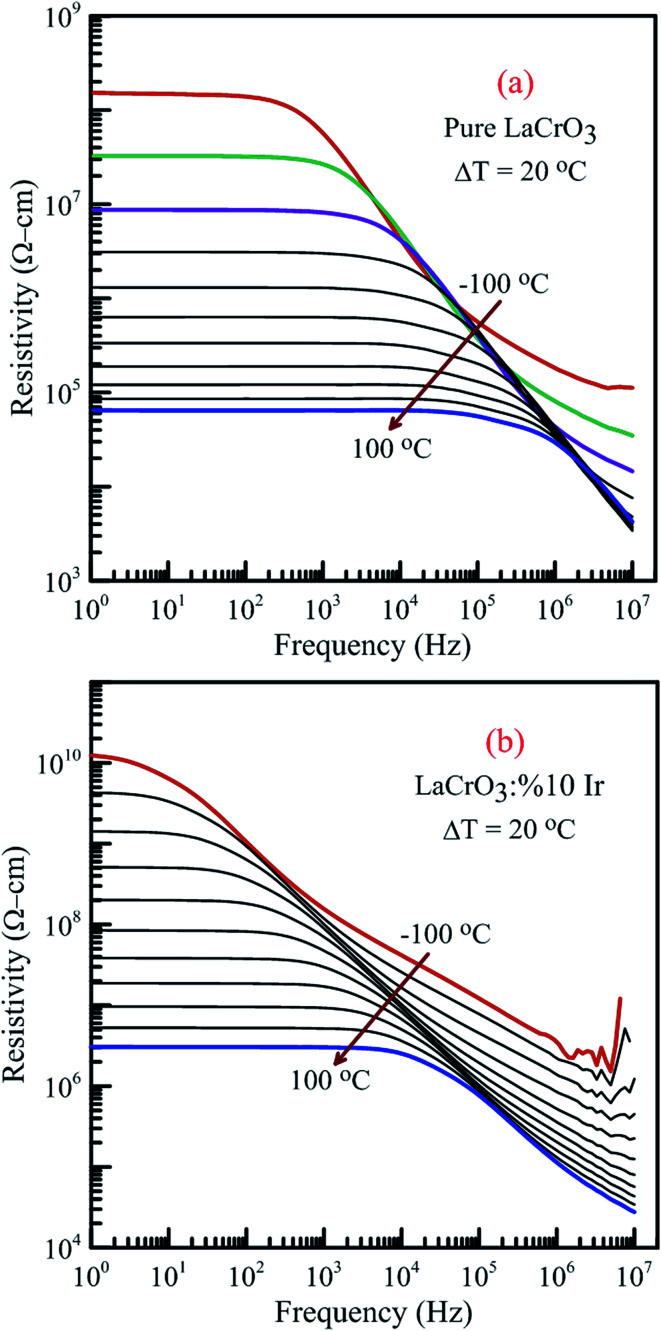
Frequency dependence of the resistivity of both samples in the temperature range from −100 °C to 100 °C with steps of 20 °C, (a) pure LaCrO_3_ and (b) LaCr_0.90_Ir_0.10_O_3_.

Fig. 15 illustrates the frequency dependence of the resistivity of both samples in the temperature range from −100 °C to 100 °C, (a) LCO sample and (b) the Ir sample doped LCO, respectively. It is well-known the total conductivity or resistivity of the sample composes of the frequency-independent dc bulk compound in low-frequency region and the frequency-dependent ac component. It is seen from Fig. 15(a) and (b) that the frequency range of the dc compound for LCO sample is wider than that of the Ir doped LCO sample at a given temperature. The resistivity decreases with increasing temperature at each frequency due to the increased mobility of oxygen vacancies or other structural defects.^17–23,33,34^

**Fig. 16:**
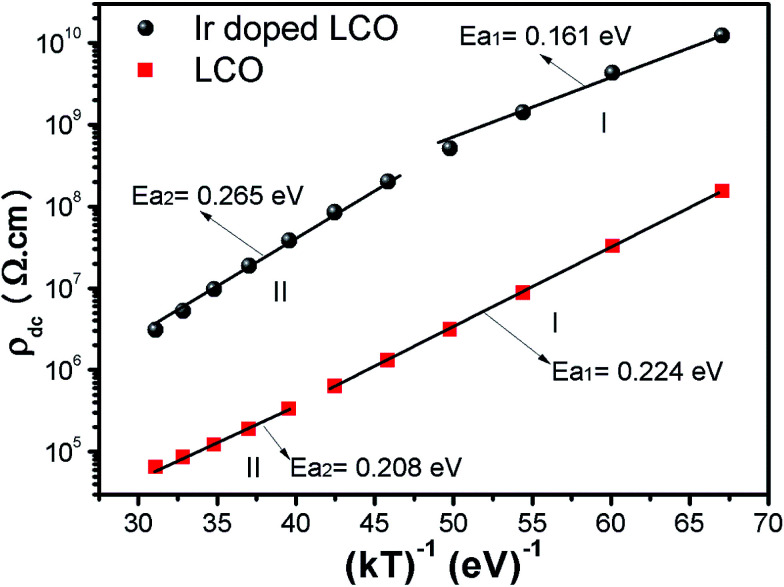
Resistivity *versus* (*kT*)^−1^ plots of both LCO and Ir doped LCO samples. The solid lines represent the fits to the experimental data.

When Fig. 15(a) and (b) have been considered for the frequency-independent dc compound of the resistivity, *ρ*_dc_, LCO sample has the values of around 1.6 × 10^8^ Ω cm at −100 °C and 6 × 10^4^ Ω cm at 100 °C, and the Ir doped LCO sample has the values of about 1.3 × 10^10^ Ω cm at −100 °C and 2.6 × 10^6^ Ω cm at 100 °C. That is, the Ir doped LCO sample has higher *ρ*_dc_ value than LCO sample at each temperature. Jiang *et al.* have also found similar results when they introduced Ba^2+^ (ionic radius 0.161 nm) into La^2+^ (ionic radius 0.136 nm). They have demonstrated that the substitution of Ba^2+^ has generated distortion in the lattice, which caused scattering of the charge carriers and hence lowered the conductivity.^65^ We believe in Ir doped LCO sample we have similar situation because ionic radii of Ir^4+^ and Ir^0^ is larger than Cr^3+^ and Cr^6+^ ionic radii. So, there will be important distortion in the LCO lattice owing to Ir substitution. In the present study, the activation energy associated with the bulk conduction has been calculated from the intercept on the resistivity axis of the frequency-independent dc component at each measured temperature. The intercept values have been used for the *ρ*_dc_*versus* (*kT*)^−1^ plot related to both samples in Fig. 16. The activation energy, cost must be paid by charge carriers to move inside the material, values of 0.224 eV at low temperature and 0.208 eV at high temperature for LCO, 0.161 eV at low temperature and 0.265 eV at high temperature for Ir doped LCO have been obtained from the slopes of the *ρ*_dc_*versus* (*kT*)^−1^ curves, respectively. The obtained activation energy values are in good agreement with previous ones obtained from the slope of *f*_max_*vs.* (*kT*)^−1^ curves.^61^ LCO has lower activation energy value at high temperature region than that at the low temperature region whereas the situation for Ir doped LCO is *vice versa*. The low activation energies show that hopping conduction is dominant mechanism for both samples. Materials with higher activation energy have higher resistivity than the materials with lower activation energy value. Therefore, our parent material LCO demonstrates less resistivity than the doped one.

## 4. Conclusions

LCO and 10% Ir doped LCO samples prepared by the solid state reaction method have exhibited different the dielectric properties from each other. The absolute dielectric constant value of both samples has increased with increasing temperature at a given frequency and it has decreased due to the doping Ir. A plateau region seems in the *M*′ *versus f* curves of LCO sample while no plateau region almost is visible in those of the Ir doped LCO one at each temperature. The imaginary *M*′′ *versus f* curves have two peaks at each temperature, one of the peaks has appeared at low frequency and other at high frequency region which shift through higher frequency region with increasing temperature. This originates from free charge accumulation at the interface with the increase of the temperature. The second peak for LCO sample shifts towards frequencies over 10 MHz at temperatures above −60 °C, that is, out measurement frequency; but, both peaks of the Ir doped LCO sample also appear in the studied frequency range, 1.0 to 10 MHz. A deviation from the semicircular shape of modulus arc of both samples has been observed with increasing temperature in high frequency region, and the semicircular arc for LCO sample have only formed at −100 and −80 °C, and the semicircular arc for the Ir doped LCO one has only formed at the other frequencies except 60, 80 and 100 °C. The absolute value of impedance curves behaves independent of frequency in low-frequency region and the frequency-dependent at high frequencies. Moreover, it increases with decreasing temperature at a given frequency, and it has been seen that the Ir doped LCO sample has higher impedance value than that of LCO sample at the same frequency and temperature. Furthermore, the activation energy values of 0.224 eV and 0.208 eV for the pure LCO and 0.161 eV and 0.265 eV for the Ir doped LCO have been obtained from the slopes of the *ρ*_dc_*vs.* (*kT*)^−1^ curves, respectively. Also the activation energies were calculated from the slopes of the *f*_max_*vs.* (*kT*)^−1^ curves and the obtained results from low frequency region were good agreement with *ρ*_dc_*vs.* (*kT*)^−1^ ones.

## Conflicts of interest

There are no conflicts to declare.

## Supplementary Material
